# Draft genome sequence of “*Candidatus* Afipia apatlaquensis” sp. nov., IBT-C3, a potential strain for decolorization of textile dyes

**DOI:** 10.1186/s13104-020-05117-y

**Published:** 2020-06-01

**Authors:** Ayixon Sánchez-Reyes, Luz Bretón-Deval, Hayley Mangelson, Alejandro Sanchez-Flores

**Affiliations:** 1 Cátedras Conacyt-Instituto de Biotecnología, Universidad Nacional Autónoma de México, Av. Universidad 2001, Chamilpa, 62210 Cuernavaca, Morelos Mexico; 2 Phase Genomics, Inc. 1617 8th Ave N, Seattle, WA 98109 USA; 3 Unidad Universitaria de Secuenciación Masiva y Bioinformática, Instituto de Biotecnología, Universidad Nacional Autónoma de México, Cuernavaca, Mexico

**Keywords:** “*Candidatus* Afipia apatlaquensis” sp. nov., Strain IBT-C3, Textile dye decolorization, Metagenomic deconvolution

## Abstract

**Objectives:**

In order to characterize a river-associated, enriched microbiome capable of degrading an anthraquinone dye from the oil blue family, as well as assessing its functional potential, we performed a taxa-specific metagenomic deconvolution analysis based on contact probability maps at the chromosomal level. This study will allow associating the genomic content of “*Candidatus* Afipia apatlaquensis” strain IBT-C3 with its phenotypic potential in the context of bioremediation of textile dyes. We anticipate that this resource will be very useful in comparative genomic clinical studies, contributing to understanding the genomic basis of *Afipia* pathogenicity.

**Data description:**

Here, we report the first draft genome sequence of “*Candidatus* Afipia apatlaquensis” sp. nov., strain IBT-C3, obtained by deconvolution of a textile-dye degrader microbiome in Mexico. The genome composite was deconvoluted using a Hi-C proximity ligation method. Whole-genome-based comparisons and phylogenomics reconstruction indicate that strain IBT-C3 represents a new species of the genus *Afipia*. The assembly completeness was 92.5% with 5,604,749 bp in length and 60.72% G+C content. The genome complement of IBT-C3 suggests a functional potential for decolorization of textile dyes, contrasting with previous reports of *Afipia* genus focused on its pathogenic potential.

## Objective

*Afipia* is a bacterial genus clustered in the *Bradyrhizobiaceae* family of the *Proteobacteria* phylum. *Afipia* species are widely known as human and animal pathogens and have been isolated from human sources or hospital water supplies. They are also considered amoeba-resisting bacteria as they can be recovered from amoebal coculture in domestic water systems, a trait related to his capacity for causing nosocomial infections. Five species of the genus have standing in nomenclature and other genospecies groups have been described in the literature [1–4]. At the time of writing, GenBank contains 27 assemblies of this genus, five of which are derived from type material. However, the majority of this taxon has been explored in a clinic context. In this study, we have deconvoluted a genome composite of a new species of *Afipia* from a mixed culture enriched with an anthraquinonic textile dye. This project aims to explore how nutritional selection by textile dyes influences microbial communities in bodies of water in the state of Morelos, Mexico. Based on genomic relationship criteria, we have named this novel taxon “*Candidatus* Afipia apatlaquensis” sp. nov., in line with its coherence inside the *Afipia* genus, but clear distinctiveness among the species already described. Analysis of the annotated genome suggests plausible molecular functions related to textile dye degradation. This is the first report of an *Afipia* genetic resource assembled from an enriched river sediment biome in a textile dye bioremediation context. Our long-term goal is to reproduce patterns of microbial dynamics that shed light on how microorganisms respond to pollution generated by the textile industry. We anticipate that this resource will be very useful in comparative genomic studies contributing to the understanding of the genomic bases that modulates environmental or pathogenic behaviors in *Afipia*.

## Data description

In order to explore the microbial diversity present in a highly polluted area of the Apatlaco river basin located in Morelos, México, four samples of sediments and surface water were taken (sites P1: − 99.26872, 18.97372, P7: − 99.2187, 18.83, P10: − 99.23337, 18.78971 and P17: − 99.18278, 18.60914) and processed as described Bretón-Deval et al. [5]. One composite sample was enriched in the laboratory with 200 mg mL^−1^ of an anthraquinone dye (Deep-Blue 35™, obtained from Monroe Chemical Company de México, S.A. de C.V, in his national commercial form) from the oil blue family. The enriched sample was incubated for 30 days at room temperature in a 10 L polyethylene batch reactor. Ten grams of the sedimented sludge in the reactor were extracted and directly crosslinked according to [6]. The sample was sent to massively parallel sequencing, proximity ligation (Hi-C) and deconvolution services from Phase Genomics, Inc. company (Seattle, USA). The sequencing of the DNA libraries yielded 11.7 Gb of pair-end reads (Data file 6) [7]. Postprocessing of the Hi-C short reads involved trimming with Trimmomatic V 0.39 [8]. The total input reads were: 144,014,062, surviving: 141,561,527 (98.30%); and clustering reads over a previous draft assembly with ProxiMeta software [6]. CheckM V 1.0.11 was used to assess genome quality stats [9]. The “*Candidatus* Afipia apatlaquensis” sp. nov., genome composite was submitted to GenBank under the BioProject: PRJNA606950 (https://identifiers.org/ncbi/bioproject:PRJNA606950) (Data file 1) [10] and was annotated with the National Center for Biotechnology Information (NCBI) Prokaryotic Annotation Pipeline [11]. In addition, the metagenome-assembled genome was annotated with KofamKOALA tool [12] in order to assign KEGG Orthologs (KO) related to decolorization of textile dyes (Data file 3) [13]. Genomic taxonomy was performed by analysis of overall genome relation indexes with Average Nucleotide Identity, Mash distance determination (Data files 2 and 4) [14, 15] and phylogenomic reconstruction with the Up-to-date bacterial core gene set (UBCG) tools (Data file 5) [16]. Table 1 presents data repositories and links for genome assembly and annotations, taxonomic descriptions and whole-genome sequence analysis.

**Table 1:** Overview of data files

Label	Name of data file	File types (file extension)	Data repository and identifier (DOI or accession number)
Data file 1	*Genome Assembly of “Candidatus* Afipia apatlaquensis*”* sp. nov.	*Fasta file* (*.fna*)	https://identifiers.org/ncbi/bioproject:PRJNA606950 [10]
Data file 2	*Overall genome relatedness index for* “*Candidatus* Afipia apatlaquensis” sp. nov., *strain IBT*-*C3 with phylogenetic Afipia neighbors.*	*Portable Document Format file* (*.pdf*)	10.6084/m9.figshare.11889015.v3 [14]
Data file 3	*Potentially relevant enzymes for textile dyes degradation encoded in* “*Candidatus* Afipia apatlaquensis” sp. nov., genome.	*Portable Document Format file* (*.pdf*)	10.6084/m9.figshare.11889012.v2 [13]
Data file 4	*Description of* “*Candidatus* Afipia apatlaquensis” sp. nov., *and Genomic features*	*Portable Document Format file* (*.pdf*)	10.6084/m9.figshare.11889018.v3 [15]
Data file 5	*Phylogenomic analysis with 92 core bacterial genes*	*Tagged Image File Format* (*.tiff*)	10.6084/m9.figshare.11889021.v2 [16]
Data file 6	*Raw reads sequence data*	*Fastq.gz file* (*.fastq.gz*)	https://www.ncbi.nlm.nih.gov/sra/PRJNA623057 [7]

## Limitations

The reported “*Candidatus* Afipia apatlaquensis” sp. nov., genome composite, was assembled from a mixed sample. However, in order to reduce bias, we apply a novel methodology that involves covalent association of nearby sequences intrachromosomally, which ensures sequences belonging to the same cell could be grouped by a physical signal.

## Data Availability

The data described in this Data note can be freely and openly accessed on https://identifiers.org/ncbi/bioproject:PRJNA606950 [10]. Data files 2 to 5 are freely accessible on Figshare (https://figshare.com/). Please see Table 1 and references [7, 10, 13–16] for details and links to the data.

## Abbreviations

EC: Enzyme commission number
G+C: Guanine–Cytosine content
HI-C: A methodology for study tridimensional structure of chromosomes
Kmer: Nucleotide subsequences of length k
KO: KEGG orthology identifier
